# “It brought hope and peace in my heart:” Caregivers perceptions on kangaroo mother care services in Malawi

**DOI:** 10.1186/s12887-020-02443-9

**Published:** 2020-12-02

**Authors:** Alinane Linda Nyondo-Mipando, Mai-Lei Woo Kinshella, Sangwani Salimu, Brandina Chiwaya, Felix Chikoti, Lusungu Chirambo, Ephrida Mwaungulu, Mwai Banda, Laura Newberry, Jenala Njirammadzi, Tamanda Hiwa, Marianne Vidler, Queen Dube, Elizabeth Molyneux, Joseph Mfutso-Bengo, David M. Goldfarb, Kondwani Kawaza

**Affiliations:** 1 School of Public Health and Family Medicine, Department of Health Systems and Policy, College of Medicine, University of Malawi, Private Bag 360, Blantyre, Malawi; 2 College of Medicine, IMCHA Project, Blantyre, Malawi; 3 Department of Obstetrics and Gynaecology, BC Children’s and Women’s Hospital, University of British Columbia, Vancouver, Canada; 4 Department of Pediatrics and Child Health, College of Medicine, University of Malawi, Blantyre, Malawi; 5 Queen Elizabeth Central Hospital, Pediatrics, Blantyre, Malawi; 6 Center of Bioethics for Eastern & Southern Africa (CEBESA), Blantyre, Malawi; 7 Department of Pathology and Laboratory Medicine, BC Children’s and Women’s Hospital, University of British Columbia, Vancouver, Canada

**Keywords:** Kangaroo Mother Care, Caregivers, Preterm Births, Gendered Roles

## Abstract

**Background:**

Kangaroo mother care (KMC) is an effective intervention for preterm and low birth weight infants. Effective implementation of KMC relies on a multidisciplinary team centering on the newborn’s caregiver, who delivers care with support from health care workers. This study explored the experiences of caregivers on the implementation of KMC.

**Methods:**

We conducted a descriptive qualitative study in the phenomenological tradition, an interpretative approach to describe the caregivers’ lived experience with KMC at four health facilities in Malawi from April and June 2019 through 10 non-participatory observations and 24 face-to-face interviews. We drew a purposive sample of 14 mothers, six fathers, three grandmothers, and one grandfather of infants receiving KMC in three secondary and one tertiary level hospitals. Data were analyzed following a thematic approach.

**Results:**

Caregivers had limited information on KMC before admission with most of the information learned from peers rather than medical professionals. Stories of positive outcomes following KMC contributed to a shift in perceptions of premature babies and acceptability of KMC as an effective intervention. Unintended consequences resulting from admission due to KMC disrupts responsibilities around the home and disrupts economic activities. Gender division of roles exists with the implementation of KMC and a mother’s support networks are crucial.

**Conclusion:**

Kangaroo mother care is feasible and acceptable among caregivers. KMC babies are described more positively with the potential to grow into strong and healthy children. KMC remains focused on the mother, which undervalues the important roles of her support network. A change in the nomenclature from kangaroo mother care to kangaroo care would include fathers and others delivering care.

## Background

Neonatal mortality remains alarmingly high in sub-Saharan Africa at a rate of 28 deaths per 1000 live births, with 75% of these occurring in the first week of life [1]. Preterm birth, birth asphyxia, infections, and birth defects are leading causes of death during the first month of life [1]. Kangaroo mother care (KMC) features sustained skin-to-skin contact between a neonate and his or her mother or other caregivers. KMC helps to keep the baby warm, facilitates exclusive breastfeeding [2], and has proved to be an effective intervention for preterm and low birthweight infants [3, 4]. KMC delivered at health facilities can reduce rates of newborn deaths by 37–40% in comparison to conventional care [3]. In comparison to conventional incubator care, KMC is more affordable and acceptable to both mothers and health workers and results in increased infant weight gain and fewer cases of hypothermia [5, 6].

Effective KMC practice involves a multidisciplinary team [7] that centers on the newborn’s caregiver to deliver the care with support from health workers [4]. In addition to skin-to-skin contact and supporting exclusive breastfeeding, a key component of KMC is the goal of early discharge from the hospital [8], which requires caregiver knowledge and acceptance [6]. Low levels of community knowledge on KMC have been previously documented [9], which could affect acceptability and compliance. Additionally, men have largely been left out of discussions around KMC, though male involvement is recognized as crucial for uptake, support, and adherence [10]. Successful implementation of KMC relies on adequate training and client-oriented care [4, 11] and peer support, particularly in understaffed, resource-constrained health settings [10, 12], thus underscoring the importance of caregiver knowledge and engagement in sustainable KMC practice.

Malawi has one of the highest rates of preterm birth in the world with 18–19.1% of all births occurring before 37 weeks of gestation [13, 14] and a third of newborn deaths are attributed to complications related to prematurity [15]. Malawi prioritized KMC to improve the quality of care for preterm and low birth weight neonates [4], and institutionalized KMC in its hospitals [16]. However, a gap remains on KMC implementation and understanding caregiver experiences. This study explored the experiences of caregivers in the implementation of KMC.

## Methods

### Design

This was a descriptive qualitative study in the phenomenological tradition, an interpretative approach that emphasizes understanding meanings people make of their experiences [17, 18], and aimed to describe the caregivers’ lived experience with KMC at four health facilities in Malawi. Our explorations of KMC are part of the larger project, “Integrating a neonatal healthcare package for Malawi”, funded as part of the Innovating for Maternal and Child Health in Africa initiative by the Canadian International Development Research Centre, Global Affairs Canada, and the Canadian Institutes for Health Research.

### Research Setting

We conducted the study at one tertiary (urban) and three secondary-level (district) hospitals in southern Malawi. Two district hospitals were government facilities while the third was a faith-based facility under a government service agreement to provide essential health services free of cost to patients. The selection of these health facilities was in liaison with the Malawi Ministry of Health and has been reported elsewhere [19]. Preterm and low birth weight (< 2500 g) newborns are admitted to KMC when determined to be in stable condition (no signs of fever, hyperthermia, weight loss, sunken eyes, protruding fontanel, or failure to feed). The timing of clinical assessment and KMC initiation varied depending on the infant’s health status and availability of staff at the time of birth.

### Recruitment

Prior to data collection, project staff briefed the clinical management teams at each of the hospitals. We employed a purposive sampling approach to select four to six caregivers of infants receiving KMC in each hospital, caregivers included mothers, fathers, and grandmothers. Our sample size was guided by the size of KMC wards (2–8 beds in each) and Guest et al’s argument that data saturation is often achieved by the 12th interview [20]. With the support of nursing officers at each hospital, we approached mothers providing KMC during their hospital stay. We prioritized caregivers of infants who had spent at least one night in the KMC ward. Those with less than five hours in the KMC ward had limited experience in the ward and were not approached for recruitment. Researchers also asked mothers if the baby’s father or other caregivers would be interested in participating in the study. Fathers rarely stayed at the hospital and were approached during the visiting hours and recruited irrespective of whether their wife had been interviewed or not. Of all women approached, two refused to take part, citing that they were busy with the care of the babies.

### Data Collection

We collected the data between April and June 2019 through non-participatory observations and face-to-face interviews of 30–60 minutes at each of the four health facilities after written consent was obtained from each participant. We piloted the topic guide at the tertiary hospital to ascertain its ability to capture the desired information and the appropriateness of the questions in local settings. A team of five Malawian researchers collected the data after intensive training in qualitative research methods. Researchers had no prior relationships with research participants. They first introduced themselves as members of IMCHA, a research group at the College of Medicine University of Malawi (CoM), and explained the study in detail. Observations were conducted in the KMC ward in each of the four hospitals following a guide covering the description of the room and infrastructure, clinical staff and patient interactions, the process of KMC practice, peer interactions, and anything else of note to the experience of KMC. Observations took on average 45–60 minutes and all observations were made during weekday day shifts.

Interviews were conducted in a private setting within the health facility, in the local language (Chichewa) and audio recorded with permission. Data collectors compiled field notes after interviews and the qualitative team had multiple discussions to reflect the process and emerging themes, to discuss the dependability of our findings and the context of our research [21]. There were no repeat interviews.

### Data Management and Analysis

Data were stored in locked cabinets at CoM and in password-protected computers with access limited to lead researchers. Audio recordings were transcribed verbatim, translated into English, and managed using NVivo 12 software (QSR International, Melbourne, Australia). Participants were assigned codes that were used throughout the data collection and management process to maintain confidentiality.

We employed a thematic approach in analyzing the data. Pilot data underwent preliminary analysis to develop the coding framework (MWK, MA in Medical Anthropology, and ALMN, Ph.D in Health Systems and Policy). Considering the exploratory approach of qualitative research and the value of shared patient stories, all data collected were analyzed including the pilot interviews. SS conducted the primary coding using the framework under the supervision of MWK and ALNM and the three discussed the data at intervals to reflect and refine the coding framework as necessary. We verified the themes against the audios to ensure that they were representative of the data.

## Results

### Participant Characteristics and Description of KMC wards

We conducted 10 days of clinical observations and 24 in-depth interviews. We interviewed 14 mothers, six fathers, three grandmothers, and one grandfather. The majority of participants were self-employed and ran small scale businesses (see Table 1). Table 2 describes KMC wards of each facility. In comparison to the tertiary hospital and the Christian Health Association of Malawi (CHAM) based facility, the KMC ward at the district hospitals was often smaller and overcrowded with beds crammed close together. Only the KMC ward in the tertiary hospital had a functioning wall heater. Facilities kept windows closed to retain heat except for one district hospital where windows remained open but they were small and high up. At district hospitals, a supporting family member accompanied mothers most of the time, while caregivers were restricted at the tertiary hospital. Only female caregivers normally visited the KMC ward in all four facilities due to privacy issues and the prevailing norm that maternity issues are confined to women. There were culturally appropriate posters of mothers carrying an infant using local cloth wrappers, information on the positioning of the baby, and benefits of KMC displayed on facility walls in the four hospitals. The KMC ward at the tertiary hospital had designated staff while nurses at the district hospitals covered both the KMC and postnatal wards. Physicians conducted ward rounds twice a week at the tertiary hospital while clinical officers oversaw all infants in the KMC and postnatal wards during their shift. In all four hospitals, clinicians were available at all times for consultations if complications emerged.

**Table 1:** Caregivers Economic Activities

Participant	Facility	Occupation
Grandmother	4	Housewife
Mother	1	Housewife
Grandmother	1	Vegetable Seller
Mother	2	Rice Seller
Father	2	Second-hand Clothes Seller
Father	3	Farmer
Father	4	Stationery Seller
Mother	1	Farmer
Mother	2	Housewife
Mother	1	Tomato Seller
Mother	2	Farmer
Mother	3	Doughnut Seller
Mother	3	Housewife
Mother	3	Tailor
Grandfather	2	Security Guard
Father	3	Informal work
Mother	2	Farmer
Mother	4	Tiler
Father	3	Waiter
Mother	4	Cooking Oil Seller
Mother	4	Sells Savories
Grand Mother	4	Housewife
Mother	4	Housewife
Father	4	Employed

**Table 2:** Facility Observations

Facility	# Beds	# Nurses^a^	Other Staff	Bathrooms and showers	Food preparation	Visiting Hours	Type of visitors
District hospital 1 (government)	4	2	Clinical officers, hospital attendants	Available	Responsibility of mothers and her family	6:30 − 7:30 12–13:00 17:00–19:00	Only women allowed
District hospital 2 (mission hospital)	8	2	Clinical officers, hospital attendants	Available; lack handwashing station in the ward	Responsibility of mothers and her family	Designated feeding times at intervals	Only women allowed
District hospital 3 (government)	2	2	Clinical officers, hospital attendants	Available	Responsibility of mothers and her family	6:30 − 7:30 12–13:00 17:00–19:00	Only women allowed
Tertiary hospital	23	2	Medical interns, physicians and pediatric specialists, hospital attendants	Available	Responsibility of mothers and her family	Designated feeding times at intervals	Only women allowed

^a^*During observations*

### Initiating and Implementing KMC

Upon arrival in the KMC ward, mothers in all four facilities were welcomed by the nurse on duty who assigned a bed and counselled mothers on the benefits and implementation of KMC. On average, counselling during KMC initiation lasted less than 10 minutes. Family members were included in the counselling if they were present. Mothers were responsible for relaying information if family members arrived later. Nurses provided medications, reminders about feeding, regularly weighed babies, and facilitated intake for new mothers; however, daily care and monitoring for danger signs were largely the responsibility of caregivers. Beyond interactions related to the provision of infant care, there was minimal interaction between health workers and caregivers observed. At district facilities, health workers were observed shouting out instructions to mothers across the room. At the four facilities, mothers brought their infants to the nurses’ station for weighing. Information on the progress of the baby was only provided when indicators such as weight and breastfeeding practice were not met at district hospitals while mothers were advised irrespective of progress on weight and breastfeeding status at the tertiary hospital.

KMC was predominantly provided by mothers with support from female relatives and other mothers in the ward. Mothers were observed assisting each other to secure babies in the KMC position by tightening each other’s wrappers. At the tertiary hospital, women also organized themselves and appointed a chairperson, who oriented newly admitted women to activities on the ward. In facilities with minimal oversight by health care workers or crowded rooms, mothers were observed taking babies off KMC to chat and engage in other activities. Family members were frequently not around in the KMC ward except for those that had been around since delivery. Men rarely entered KMC wards and met their wives outside of the ward.

### Caregiver Knowledge and Acceptance of Kangaroo Mother Care

Most caregivers reported minimal previous knowledge of KMC and shared that they only learned about the practice when their low birth weight infants were admitted.

*“I never heard about babies being put on the chest, but this was my first time to see a baby being put on the chest as it was my wife doing it, so I see it with my eyes, but at first, I didn’t know what goes on.” Father at a district hospital*.

*“Those in the village that have never experienced the kangaroo mother care can be surprised by putting the child on the chest.” Father at the tertiary hospital*.

KMC could be a source of fear, especially for those practicing for the first time and had no prior knowledge. Some were scared of harming their newborns due to the small size, fragility, or new ways of holding the baby. Caregivers shared that skin-to-skin contact on the chest was different than the typical practice of carrying children on the back.

*“You see, we are used to putting our babies at our back. This time, we’re doing the opposite whereby we are putting the babies on our stomach. This means it will be different in the way we used to do.” Mother at the tertiary hospital*.

*“I was scared to put a baby on a KMC position while I am sleeping. I was thinking about how am I going to be turning while asleep? And since we were told not to be turning while sleeping, I was so worried. Mother at a district hospital*.

However, after an explanation from health workers on KMC and witnessing lives saved in the KMC ward, caregivers shared that they grew to appreciate its benefits.

*“At first I was worried because my wife told me that the baby was very small and she doubted if the baby will survive, but later on after seeing other women going through the same and that their babies are surviving, I felt happy that my baby’s life is going to be saved. You know, this is a good intervention…It’s now three weeks since the baby was initiated on the kangaroo, the baby was so small but now it is growing, it is gaining weight and now I am happy…I know my baby could have easily died without this intervention but now I know there are very high chances that my baby will make it and we will be discharged from the hospital.” Father at a district hospital*.

*“Nurses explained very well and encouraged us to adopt the initiative by telling us advantages of kangaroo mother care and how is done…He started by telling me that “Grandma, we want your grandson to survive.” So from that statement, I knew that every initiative that would be put in place would be for the survival of my grandson… You know when doctors or these nurses come to your bed you feel like your grandson is also considered important by them and gives a sense of hope. They send a message to us that they are hoping for life on our patient.” Grandmother at the tertiary hospital*.

### Changing Practices and Perspectives of Preterm Infants

The few participants who had prior knowledge about KMC stated that they had learned from other community members with experiences of KMC with their children. Growing awareness of KMC practice was driven through peer networks rather than prenatal counselling. These were often positive stories about survival. A grandfather from a district hospital visiting his daughter and grandson recounted what his friend had told him and remarked how the premature infant has grown into a strong young man. He laughingly said,

*“There is a friend I pray with…who told me not to worry. He said, “my grandchild was also born prematurely’. They followed the rules that the doctors told them and seeing the child [now], he can beat me!... I cannot try him because I see his hand is very fat” Grandfather at a district hospital*.

Another said,

*“There are children at our home who went through the same but now they are grown up now.” Father at the tertiary hospital*. Witnessing survival of low birth weight infants with KMC supported shifting perceptions around premature newborns. Previously, preterm babies were highly stigmatized and viewed as barely human. Families were ashamed of their small babies and shared that women would previously hide them in the house, beside a fire, or wrapped up for warmth. The introduction of KMC has supported changing perceptions of preterm babies from fated to die, to able to survive if properly managed.

*“Premature babies were called elephants, small on one side and big on others. Babies were wrapped around and kept for a month, and babies would gain weight... If there were not enough wrappers, the babies would die.” Grandmother at a district hospital*.

*“In the past, people could say [that] preterm and underweight babies are not human beings and not worth living, but now people are accepting it and they do allow their wives or family members to put their babies on kangaroo…” Father at a district hospital*.

*“People just say it is one of the modern ways to save lives of babies” Mother at a district hospital*.

### Perspectives on Unintended Consequences of KMC Admission

Though caregivers often spoke about KMC positively, they also reported the unintended consequences that facility-based KMC exerts on families. These include changes in responsibilities and the impacts that prolonged hospitalization can have on livelihoods.

#### Change in responsibilities

The admission of mothers and their newborn(s) into the KMC ward can create changes in responsibilities around the home, including around the care of other children. Many of the men interviewed shared challenges in the need to care for the home at the same time as ensuring that his wife and baby were well taken care of at the hospital. Mothers sometimes expressed concern about the care of her home and shared that she missed them during their separation. Due to the unexpected hospital admission for preterm births, families often felt unprepared and concerned that a prolonged hospital stay would strain their household capacities to support caring for other children left at home.

*“As she is here at the hospital, we are worried because a home is nothing without a woman, even though there is someone taking care of it, and at the moment, I am taking care of two places” Father at a district hospital*.

*“Yes I am worried, I miss my home and my kids. I don’t know if things are being properly taken care of. When I am at home, I make sure everything is in order, so now I am just looking forward to being discharged, for me to go home and take care of my children and other things at home, but the thing is…. I am not sure of the exact time when I will be discharged from the hospital.” Mother at a district hospital*.

#### Economic Activities

Families expressed concern over the disruption in their livelihoods due to lengthy hospital admission. Most participants were informally employed and depended on their day-to-day, small-scale business activities, and/or subsistence farming for survival (see Table 2**)**. Disruptions affected mothers admitted to the hospital as well as her family.

*“I had prepared for this visit to stay about four months until my daughter-in-law delivers…This is the first month of my stay here…. However, I don’t know when my grandson is going to be ok. My worry therefore is that should the stay surpass the planned four months. I am afraid I may not have time to grow crops and definitely, our family might be at risk of hunger…” Grandmother at the tertiary hospital*.

The economic challenges also applied to those that were formally employed;

*“They gave me only seven days as a holiday and I am worried they may terminate my contract should I stay here longer than expected.” Father at a district hospital*.

While some caregivers described the process of KMC as inexpensive compared to other medical services, others highlighted the economic demands of supplies required: bedding linens, toiletries, hats for infants, and the cloth wrap to perform KMC. In addition, hospitals provided minimal, if any, food. At the same time that their livelihoods were constrained, caregivers expressed that costs related to KMC challenged continued KMC practice.

*“You must have enough sheets, you should have a shawl, socks, and a hat and when you are putting the baby between the breasts…. Hence, the lack of money to buy those things, considering you have to take care of both the family and the baby, causes some to just say they will just figure it on their own at home and ditch this program.” Mother at a district hospital*.

### Gender Roles in KMC

#### Mothers’ role

Caregivers highlighted that mothers play a central role in KMC. Mothers felt that KMC and caring for the daily needs of their baby rested on them. The care of their infants in the KMC ward was an extension of their gendered role as mothers. Additionally, some mothers highlighted their roles in monitoring their infant’s well-being. Observations showed nurses informing mothers on how to take care of their babies during KMC including feeding, clothing, positioning, and education on danger signs such as jaundice, respiratory problems, low and high temperatures, and babies turning blue. Nurses counselled mothers to contact them if complications arise.

*“As a mother, I have to take care of my baby as a mother is supposed to do, I have to show motherly love…Since I am here taking care of my baby on the kangaroo, I have to make sure I am always keeping my baby warm, I am feeding baby, I wipe my baby with a warm clean cloth to keep the baby warm…” Mother at a district hospital*.

#### Fathers’ role

While mothers were seen as central to providing care during KMC, male family members were also seen to play important roles. Fathers and grandfathers were a critical link between the home and health facility. They shared that their role was to provide for food and supplies during their stay at the hospital as well as to organize for a female family member to come and assist the mother. Men also shared that they offered emotional support to their partners.

*“As the father, I have the role to make sure the baby is well taken care of together with the mum, and I have to provide whatever is needed for both of them, as the health personnel only assist us in medical terms but I have to provide like food and whatever they may need…. I have to encourage the mother to always keep the baby on her chest and if she is to attend to other things, I have to find someone to help her” Father at a district hospital*.

Though men in the study expressed willingness to be more involved in KMC, they acknowledged the limitations due to gender norms and conceptualization of the ward as a female space. Culturally, skin-to-skin contact is considered a woman’s role.

*“I don’t think the health personnel can allow me to be a full-time guardian here as these are female wards so I cannot be sleeping here with the women…and another thing is that it is not easy for me to put the baby on the chest as I am a man…. As by tradition, this is taken as a woman’s role and if people see me doing this, I think they will start talking about us. In my life, I have never seen a man doing this, I mean, carrying a small baby on his chest.” Father at a district hospital*.

*“If done at home, we (husbands) can easily be assisting. Here at the hospital, it’s not easy…Maybe it’s the way men are socialized in Malawi because I have never seen a man putting a baby on the kangaroo, I only see women.” Father at the tertiary hospital*.

#### Other Family Support

Grandmothers, including maternal grandmothers if the mother was young and paternal grandmothers (mother-in-law), also played a critical role in supporting KMC. Their responsibilities included implementing KMC when a mother is resting, or not well, as well as helping with personal care of the baby and mother. Observations revealed that grandmothers supported in the case of twins and when the mother delivered by caesarean section. Mothers shared that they were very appreciative of the support received.

*“I have a lot of responsibilities. I think the first one is to help the mother is doing the kangaroo mother care. When she wants to rest, the baby is put on me and I do exactly what she does except breastfeeding. The second thing is to see her doing everything accordingly as told by the doctors… Also, I think it is my responsibility to encourage her that things are going to be ok.” Grandmother at the tertiary hospital*.

*“For me, I think the factor that makes it easy for me to do kangaroo mother care is that I have someone who helps me. That is my mother. Had it been that I am doing it alone, life could have been very difficult, but the good thing is mum is here and she always offers her help.” Mother at a district hospital*.

## Discussion

In this study, we explored caregiver experiences and perspectives of the implementation of KMC. We found that though previous knowledge of KMC varied, the interviewed caregivers largely had very limited information prior to admission and background information was learned from peers rather than medical professionals. Growing community awareness of success stories in the survival of low birthweight babies supported a shift in perceptions of premature babies and has led to the acceptability of KMC. However, KMC has unintended consequences on responsibilities around the home and can disrupt economic activities, especially with prolonged hospitalization. Gender roles exist with the implementation of KMC and a mother’s support networks are crucial.

Our findings that most caregivers had minimal knowledge of KMC prior to admission resonates with other studies on the scale-up of KMC in sub-Saharan Africa [9, 22]. A study on KMC implementation in South Africa also found that women were educated about KMC upon hospital admission, not during prenatal counselling [22]. The antenatal period is a lost opportunity for engaging women about preterm delivery and interventions like KMC [10], especially in Malawi with a high rate of prematurity [23]. Inadequate prenatal education regarding prematurity leaves mothers unprepared with limited coping skills, which burdens health workers in busy maternity wards [22]. A systematic review of barriers and facilitators of KMC practice including 103 studies from around the world found that low caregiver knowledge on KMC negatively impacted the implementation and acceptance of KMC [24].

Participants in our study shared that the introduction of KMC changed perceptions regarding premature babies, which builds on earlier findings from a Malawian community-based study that reported a shift in rural areas towards a positive perception of KMC and premature infants [10]. Preterm infants have been previously seen as deformed or associated with HIV/AIDS [25], a bad omen and could not survive [6, 12], a result of witchcraft or a polygamous marriage [26]. Other studies previously documented that mothers may be ashamed and less motivated to care for their preterm babies [27], though this did not emerge in our interviews perhaps due to recruiting caregivers from KMC wards who had accepted the practice.

Similar to previous studies on the scale-up of KMC in sub-Saharan Africa, implementation of facility-based KMC was impeded by the limited support mothers received [4, 11, 24, 28], which was exacerbated by staff shortages [6, 24, 29]. This appeared especially evident in district hospital settings in our study where nurses and clinicians covered multiple wards. Our study found an overreliance on a mother’s support network to care for herself and her newborn while at the hospital. Lengthy hospitalizations were disruptive to the home and livelihood for mothers, female relatives who supported her care at the hospital, and male relatives who provided food and supplies. Our findings on the relevance of family members in the delivery of KMC underscores what was reported by two systematic reviews that the absence of social support was a barrier to KMC [24, 27]. Not only are husbands and mothers-in-law important household decision-makers that influence the uptake of KMC [6, 11, 12], they are also the backbone of her support network. While KMC is recommended as a feasible and cost-effective intervention for resource-limited settings in comparison with conventional incubator care, this research highlights that the burden of care may be shifted onto mothers and her family to fill the gap created by a lack of human resource [24, 30].

Our study revealed highly gendered patterns of family support. While fathers reported willingness to practice KMC as in other studies [27], cultural norms around childcare and lack of privacy in the KMC ward impeded their involvement. A previous review found that overcrowding, noisy environment, and lack of privacy were barriers to KMC implementation [24] and the gender dimensions of this emerged in our study. The current setting of KMC wards was an open space, which does not allow for privacy. Due to privacy concerns, men were not allowed in the KMC ward and shared that while they were willing, they would not want to practice skin-to-skin contact with a newborn where others could see since it is a violation of gender norms.

With challenges around facility-based KMC, strengthening community-based KMC may be a potential solution. While early discharge from the hospital to continue KMC practice at home would minimize disruptions of a long hospital stay and reduces infection risks, it is largely neglected and challenged by poor follow-up networks [23, 31]. Poor follow-up in the community and the need for referral to hospitals for neonatal complications instead of the capacity to address locally at health centres meant that health providers were often conservative in discharge policies [23]. More research is needed on community-based KMC, including what health system strengthening is needed to support it. [23] Additionally, research should address the effective promotion of community-based KMC, considering that household chores and care of the other children may hinder effective implementation [28]. Other areas should identify the best ways of raising awareness in community settings, such as through religious and community leaders [10] and grandmothers [28]. Community-level engagement can also be promoted through sensitization of KMC practice during prenatal counselling and encouragement for birth preparedness in case of preterm delivery, especially in places where there are high rates of prematurity like Malawi. The current dissemination of information is through peer networks, which could be strengthened by standardized messages delivered by health professionals.

### Strengths and Limitations

The strengths of this study include interviews with different groups of caregivers across four hospital sites in Malawi to gain a broad perspective of KMC and interviews were complemented by observations in the wards. However, because the study was based at health facilities, we were more likely to hear positive rather than the negative aspect of women who self-discharged early or refused the intervention altogether. Additionally, the availability of men in the maternity unit was sparse and this may have limited men’s participation, thus more research with men in community settings is recommended. Although our sample size was relatively small, our rich qualitative research sheds light on the experiences that may be explored further. More research is recommended on the quality of life for mothers or other primary caregivers while providing KMC to understand the challenges they face as well as research specifically interviewing mothers who discharge early.

## Conclusions

Kangaroo mother care was described as a feasible and acceptable intervention by caregivers interviewed in our study, which is positive because a body of evidence has shown that KMC can reduce the mortality and morbidity rate in low birth weight and preterm infants. Among the caregivers in our study, conceptualizations of preterm infants and KMC have become so intertwined that their meanings have begun changing in the context of each other and the terms were sometimes used interchangeably. Instead of the stigma of a preterm infant, KMC babies were described more positively with the potential to grow into strong and healthy children. However, our study found that KMC is currently focused on the mother, which undervalues the important roles of her support network, the potential for other family members to practice, and the need to strengthen health systems to adequately support caregivers. To broaden the scope and the synergistic elements needed for effective scale-up, we recommend a reconceptualization from kangaroo mother care to kangaroo care. Moving beyond the mother and infant dyad, our research in a low-resource setting reveals a whole community of care involved in sustainable practice.

## Data Availability

The datasets used and/or analysed during the current study are available from the corresponding author on reasonable request.

## Abbreviations

CHAM: Christian Health Association of Malawi
CoM: College of Medicine
CoMREC: College of Medicine Research and Ethics Committee
KMC: Kangaroo Mother Care
